# Characterization of FRM-36143 as a new γ-secretase modulator for the potential treatment of familial Alzheimer’s disease

**DOI:** 10.1186/s13195-016-0199-5

**Published:** 2016-08-30

**Authors:** Jean-François Blain, Matthew G. Bursavich, Emily A. Freeman, Lori A. Hrdlicka, Hilliary E. Hodgdon, Ting Chen, Don E. Costa, Bryce A. Harrison, Sudarshan Kapadnis, Deirdre A. Murphy, Scott Nolan, Zhiming Tu, Cuyue Tang, Duane A. Burnett, Holger Patzke, Gerhard Koenig

**Affiliations:** FORUM Pharmaceuticals Inc, 225 2nd Avenue, Waltham, MA 02451 USA

**Keywords:** Familial Alzheimer’s disease, γ-Secretase modulator, Presenilin, Mutations, Aggregation, Drug-like properties, Aβ, Brain, CSF, Cerebrospinal fluid

## Abstract

**Background:**

Familial Alzheimer’s disease (FAD) is caused by mutations in the amyloid precursor protein (APP) or presenilin (PS). Most PS mutations, which account for the majority of FAD cases, lead to an increased ratio of longer to shorter forms of the amyloid beta (Aβ) peptide. The therapeutic rationale of γ-secretase modulators (GSMs) for Alzheimer’s disease is based on this genetic evidence as well as on enzyme kinetics measurements showing changes in the processivity of the γ-secretase complex. This analysis suggests that GSMs could potentially offset some of the effects of PS mutations on APP processing, thereby addressing the root cause of early onset FAD. Unfortunately, the field has generated few, if any, molecules with good central nervous system (CNS) drug-like properties to enable proof-of-mechanism studies.

**Method:**

We characterized the novel GSM FRM-36143 using multiple cellular assays to determine its in vitro potency and off-target activity as well as its potential to reverse the effect of PS mutations. We also tested its efficacy in vivo in wild-type mice and rats.

**Results:**

FRM-36143 has much improved CNS drug-like properties compared to published GSMs. It has an in vitro EC_50_ for Aβ_42_ of 35 nM in H4 cells, can reduce Aβ_42_ to 58 % of the baseline in rat cerebrospinal fluid, and also increases the non-amyloidogenic peptides Aβ_37_ and Aβ_38_. It does not inhibit Notch processing, nor does it inhibit 24-dehydrocholesterol reductase (DHCR24) activity. Most interestingly, it can reverse the effects of presenilin mutations on APP processing in vitro.

**Conclusions:**

FRM-36143 possesses all the characteristics of a GSM in terms of Aβ modulation Because FRM-36143 was able to reverse the effect of PS mutations, we suggest that targeting patients with this genetic defect would be the best approach at testing the efficacy of a GSM in the clinic. While the amyloid hypothesis is still being tested with β-site APP-cleaving enzyme inhibitors and monoclonal antibodies in sporadic AD, we believe it is not a hypothesis for FAD. Since GSMs can correct the molecular defect caused by PS mutations, they have the promise to provide benefits to the patients when treated early enough in the course of the disease.

## Background

Alzheimer’s disease (AD) is an age-related and chronic neurodegenerative disease that manifests itself by a progressive cognitive decline followed by gradual personality changes that ultimately result in death, typically 3 to 9 years after diagnosis [1, 2]. Neuropathologically, the disease is characterized by the presence of senile plaques, neurofibrillary tangles, as well as neuronal and synaptic loss in regions involved in learning and memory such as the hippocampus and cortical regions [3]. Senile plaques are composed of beta amyloid (Aβ) [4–6], which is a proteolytic fragment of the amyloid precursor protein (APP) produced from sequential cleavages by the β-site APP-cleaving enzyme (BACE) and γ-secretase (GS). APP is first cleaved by BACE to generate a 99 amino acid fragment (C99) [7], which is then cleaved by GS [8, 9] to generate Aβ peptides of different lengths. The most abundant product from these cleavage events is Aβ_40_, but a relatively minor species, Aβ_42_, is deposited predominantly in AD brains [10] and found together in the core of plaques with Aβ_43_ [11].

γ-Secretase is a protein complex composed of four different subunits: presenilin (PS), nicastrin (Nct), anterior pharynx-defective 1 (Aph-1), and presenilin enhancer 2 (Pen-2) in a 1:1:1:1 stoichiometry [12], with PS forming the catalytic subunit of GS [8, 9]. It cleaves type I transmembrane proteins and has more than 90 reported substrates [13], of which APP and Notch are the best characterized.

Mutations in the substrate APP [14] and in the GS component PS1/2 [15, 16] have been reported to cause familial AD, with the majority leading to an increase in the ratio of Aβ_42_:Aβ_40_ [17–19]. It is now well accepted that this increase is not due to an increased production of Aβ_42_ but rather to a reduction in the efficiency of GS to process its substrate (for a review see [20]). The genetic evidence supported the amyloid hypothesis of AD [21–23], which has since been refined to suggest that a form of soluble Aβ, rather than the Aβ aggregates found in amyloid plaques, is responsible for neurotoxicity [24].

Because of this accumulation of evidence, the pharmaceutical industry began developing compounds targeting GS to prevent the production of these toxic Aβ peptides, hoping that it could ultimately delay the progression of AD symptoms. Initially, γ-secretase inhibitors (GSIs) were developed but have since been abandoned as a treatment for AD because of mechanism-based toxicities. This issue has been exemplified in a Phase 3 clinical trial with semagacestat [25] as well as a Phase 2 trial with avagacestat [26]. Weight loss, skin cancers, and infections were among the side effects reported, but most striking was the cognitive decline reported in the semagacestat trial [25, 27, 28]. Inhibition of Notch processing is likely to be the cause of most of these side effects, but the memory decline could also be due to the inhibition of EphA4 processing (involved in dendritic spine formation) [29] or the accumulation of C99 in the membrane [30].

Different classes of molecules termed γ-secretase modulators (GSMs) were discovered that could shift the processing of Aβ from longer toxic forms to shorter nontoxic forms [31]. Interestingly, these molecules did not prevent the cleavage of Notch and other substrates, thus making them potentially devoid of on-target related side effects. The first generation of GSMs was based on nonsteroidal anti-inflammatory drug (NSAID) derivatives, but further development has since produced molecules with better potency that are more attractive for the treatment of AD [20, 32].

In the present study, we report the characterization of FRM-36143, which came from the optimization of a novel structural class of GSMs. We show that it is an orally active modulator in mice and rats and possesses good pharmacokinetic properties; it reduces longer toxic Aβ species while increasing shorter nontoxic forms. It is devoid of on-target Notch toxicity as well as off-target effects on the cholesterol biosynthetic pathway, specifically 24-dehydrocholesterol reductase (DHCR24), as was reported for the Eisai GSM E2012 [33]. In summary, we believe this compound is an effective new agent for the potential treatment of familial Alzheimer’s disease (FAD).

## Methods

### Compound

FRM-36143, (*R*)-5-(benzofuran-2-yl)-3-(6-methoxy-5-(4-methyl-1H-imidazol-1-yl)pyridin-2-yl)-5,6-dihydro-4H-1,2,4-oxadiazine, was synthesized according to literature procedures (Bursavich MG, Harrison BA, Costa DE, Hodgdon HE, Freeman EA, Hrdlicka LA, Kapadnis S, Moffit J, Murphy DA, Patzke H, Tang C, Wen M, Burnett DA, Koenig G, Blain JF. Design, synthesis and evaluation of a novel series of oxadiazine Gamma Secretase Modulators for familial Alzheimer’s disease, Submitted). It was used at >98 % purity in all assays.

### NotchΔE and luciferase reporter constructs

The wild-type CBF1 binding element was cloned in front of the SV40 promoter-driven luciferase reporter construct of pGL3pro (Promega, Madison, WI, USA) using the following oligonucleotides:

- CBF1 Fw (5’→3’): GATCTGGTGTAAACACGCCGTGGGAAAAAATTTATG
- CBF1 Rv (5’→3’): GATCCATAAATTTTTTCCCACGGCGTGTTTACACCA

Oligonucleotides were phosphorylated using T4 polynucleotide kinase and annealed (by heating to 90 °C and cooling to room temperature in a buffer containing 0.1 M NaCl). Plasmid pGL3pro was cleaved with *Bgl*II and dephosphorylated using alkaline phosphatase. The oligonucleotide pair was then ligated using T4 ligase with the linearized plasmid DNA. A clone containing 6 copies of the CBF1 element in front of the SV40 promoter was chosen. The 6xCBF1-RE/SV40 promoter was excised from pGL3 using *Xho*I and *Hin*dIII, and the fragment transferred to the pGL4.17 [luc2/Neo] vector (Promega) was opened with the same restriction enzymes.

The full length human Notch in plasmid pCMV6-XL5 (Origene, Rockville, MD, USA) was used to amplify a truncated form of Notch (NotchΔE) containing the 20-amino-acid (a.a.) signal sequence followed by 5 a.a. (PPAQL) in front of the transmembrane domain (a.a. 1735–1747) and intracellular domain (a.a. 1748–2555). The primers used were as follows:

- NotchΔE Fw (5’→3’): TTTTGAATTCGCCATGCCGCCGCTCCTGGCGCCCCTGCTCTGCCTGGCGCTGCTGCCCGCGCTCGCCGCACGAGGCCCGCCGGCGCAGCTGCACTTCATGTACGTG
- NotchΔE Rv (5’→3’): TTTTCTAGAAGCTATTACTTGAACGCCTCCGGGATGCGCGC

The resulting DNA fragment was digested with *Eco*RI and *Xba*I and ligated into the pCMV6-XL5 vector (Origene) digested with *Eco*RI plus *Xba*I. Plasmid DNA corresponding to pCMV6-XL5 NotchΔE was cut with *Xba*I, blunted with Klenow polymerase, and the cDNA insert excised with *Eco*RI. This 2500-bp fragment was then ligated into pSG5 (Stratagene, Agilent, Santa Clara, CA, USA) opened with *Bam*HI/blunt and *Eco*RI. A point mutation was inserted to obtain Met28Val in order to eliminate a potential start codon. Finally, the cDNA insert of pSG5 NotchΔE M28V was transferred to plasmid pCMV6-XL5 by digesting with *Bgl*II, filling the protruding ends in with Klenow polymerase, and then digesting with *Eco*RI. The 2500-bp DNA fragment was then ligated into pCMV6-XL5 DNA that was opened with *Xba*I, the protruding ends filled in with Klenow, and cut with *Eco*RI.

### Cell-based assays

#### H4 cells

Human neuroglioma H4 cells stably expressing wild-type human APP751 were used for this assay [34]. Briefly, cells were plated at 15,000 cells/well in 96-well plates and left to settle for 6 h (37 °C; 5 % CO_2_). Cells were then washed three times with Pro293™ chemically defined medium (Lonza, Walkersville, MD, USA) followed by the addition of the compound (0.3 % final DMSO). Plates were incubated overnight, and the supernatant was removed for quantification of Aβ peptides by sandwich enzyme-linked immunosorbent assay (ELISA) (see below). Cytotoxicity was evaluated using Cell-Titer 96 W AQueous One Solution Cell Proliferation Assay (MTS Assay, Promega) according to the manufacturer’s protocol.

#### Primary cortical neurons

Primary cultures were established from the neocortex of E17 CD1 mouse embryos (Charles River Laboratories, Wilmington, MA, USA). Following tissue dissection and trituration, the cultures were suspended in Neurobasal® medium (Invitrogen, Carlsbad, CA, USA) supplemented with 10 % horse serum (Sigma-Aldrich, St. Louis, MO, USA) and 520 μM l-glutamine. Cells were plated at 50,000 cells/well in 96-well poly-d-lysine-coated plates. Following incubation at 37 °C and 5 % CO_2_ for 4 h, the plating medium was exchanged with Neurobasal® medium supplemented with 2 % B-27® (Invitrogen), 520 μM of l-glutamine, and 1 % penicillin–streptomycin. An assay was performed eight days after plating (DIV8) by replacing half of the medium with fresh medium containing compound (0.1 % final DMSO concentration). Cultures were incubated overnight for analysis of Aβ peptides by sandwich ELISA and cytotoxicity by MTS assay.

#### Notch reporter assay

HeLa cells stably transfected with the 6xCBF1-Luc reporter were transiently transfected with the NotchΔE construct (4 μg DNA/10^6^ cells) using Lipofectamine 2000 (Thermo Fisher, Waltham, MA, USA) in 6-well plates. The day after transfection, cells were trypsinized and re-plated in black 96-well clear bottom plates. Cells were left to attach for 4 h and compounds added for overnight treatment. At the end of treatment, the media was replaced with phosphate-buffered saline (PBS,with Ca^2+^/Mg^2+^), Steady-Lite Plus reagent (Perkin Elmer, Hopkinton, MA, USA) was added, and the plate incubated at room temperature for 10 min. Chemiluminescence was then read on an EnVision plate reader (Perkin Elmer).

#### Desmosterol assay

HepG2 cells were grown in Eagle's minimal essential medium (EMEM) containing 10 % fetal bovine serum, penicillin (50 U/mL), and streptomycin (50 ug/mL) and plated at a density of 1 million cells/well in 6-well plates for the experiment. Four to five days after plating, the cells were washed with Hank’s balanced salt solution (HBSS), and compound treatment applied for 2 h in HBSS. At the end of the treatment, cells were collected, washed with PBS, and pelleted. Sterols were extracted from the cell pellet by adding 100 μL PBS and 3 mL ethylacetate (containing an internal standard of 1 mg/mL d-cholesterol). Tubes were vortexed for 20 min and centrifuged at 2000 × *g* for 20 min at room temperature. The organic phase was dried under a stream of nitrogen at 40 °C and the samples reconstituted in 65 % methanol. Liquid chromatography-tandem mass spectrometry (LC-MS/MS) analysis was performed using a Shimadzu 20-series UFLC (Shimadzu, Kyoto, Japan) and an API 5500 (Applied Biosystems, Foster City, CA, USA) with a Hypersil GOLD column (100X2.1 mM; Thermo Fisher).

### ELISA for Aβ species

Aβ peptide levels were quantified by sandwich ELISA using anti-Aβ_38_, anti-Aβ_40_, anti-Aβ_42_, (BioLegend, Dedham, MA, USA) or anti-Aβ_37_ for the capture and 4G8-horseradish peroxidase (HRP; BioLegend) for detection. When measuring Aβ_37_ and Aβ_38_, 4G8-HRP was added to the sample for overnight incubation, whereas it was added after the overnight incubation for 1 h for Aβ_40_ and Aβ_42_ measurements.

For cell-based assays, freshly collected samples of cultured cell supernatant were added to the plates and incubated at 4 °C for about 24 h. Detection was performed using SureBlue 3,3’,5,5’-tetramethylbenzidine (TMB) peroxidase substrate (KPL, Inc., Gaithersburg, MD, USA) and the plates read on a SpectraMax M5e microplate reader (Molecular Devices, Inc., Sunnyvale, CA, USA). GSM-treated samples were normalized to samples treated with DMSO alone (100 %) and 5 μM GSI, DAPT (0 %; Sigma-Aldrich). EC_50_ values were calculated from values reported as percentage of DMSO using nonlinear regression, based on a sigmoidal dose–response (variable slope) model.

For in vivo assessment of compound efficacy, brain tissue and cerebrospinal fluid (CSF) were collected, snap frozen in liquid nitrogen, and stored at −80 °C. Brain hemispheres were homogenized in 0.6 % diethylamine (DEA) in 50 mM NaCl containing protease inhibitor cocktail (cOmplete mini, EDTA-free, Roche) using sonication (Branson) at 23 % amplitude for 30 s. Homogenates were spun at 227,000 × *g* for 25 min at 4 °C. Supernatants were diluted fivefold in PBS-T (0.05 % Tween-20) containing 0.67 % BSA and added to the ELISA plate. For CSF, samples were diluted threefold in the PBS-T/BSA buffer. Detection was performed using the SuperSignal™ ELISA Femto substrate (Thermo Fisher), and luminescence was read on an EnVision plate reader (Perkin Elmer).

### Aβ aggregation assay

Aβ peptides (AnaSpec, Fremont, CA, USA) were dissolved at a concentration of 1 mg/mL in hexafluoroisopropanol (HFIP). Peptides were then mixed at different molar ratios. HFIP was evaporated in a SpeedVac without heating for 15 min. Peptides and mixtures were kept on ice and reconstituted in 50 mM Tris–HCl, 1 mM EDTA. Peptides (final concentration: 10 μM) were added to thioflavin T (final concentration: 2.5 μM; AnaSpec) in a black 96-well plate. Aggregation was monitored on a SpectraMax M5 plate reader (Molecular Devices, Inc.) at a constant temperature of 25 °C using excitation/emission of 440 nm/480 nm. Readings were recorded in triplicate every 10 min for a period of 18 h.

### Acute treatment studies in mice and rats

Mice and rats were maintained on 12/12 h light/dark cycle with food available ad libitum. Drug treatment was prepared in a vehicle of 1 % carboxymethylcellulose (CMC):Tween80 (99.5:0.5). Twelve-week-old wild-type male mice (129S6, Taconic Biosciences, Hudson, NY, USA) or rats (Wistar, Charles River Laboratories) were administered compound p.o. without prior fasting and euthanized by CO_2_ asphyxiation at specified times post-dosing. After extraction of the brain, the olfactory bulb and hindbrain were removed and the cerebral hemispheres separated (mice) or cut in 4 pieces (rats). The cerebellum was removed for bioanalysis of the compound. CSF was collected from the cisterna magna and blood from a cardiac puncture.

### Bioanalytical methods

Cerebellums were homogenized in a Mini-BeadBeater (Biospec Products, Inc., Bartlesville, OK, USA). Brain samples, plasma, and CSF were prepared for LC-MS/MS by precipitating proteins with acetonitrile and vacuum filtration in the presence of an internal standard. Standards were prepared in the corresponding matrix. FRM-36143 was resolved by HPLC (Shimadzu) using a reverse-phase C18 Kinetex column (30X2.1 mM; Phenomenex, Torrance, CA, USA). Following separation, the column effluent was introduced into a triple quadrupole mass spectrometer (API 5500, Applied Biosystems), optimized for detection of FRM-36143 and using multiple reaction monitoring with mass transition of 390.3 > 215.2.

### Protein binding

The unbound fraction in brain homogenate (*f*_u,b_) was determined in triplicate in the Rapid Equilibrium Dialysis device (8000 Da molecular weight cut-off membrane, Thermo Scientific/Pierce Biotechnology, Rockford, IL, USA). Briefly, FRM-36143 stock solution (0.1 mg/mL in acetonitrile) was added to fresh mouse brain homogenate (1:4 dilution with PBS) to achieve a final concentration of 1 μg/mL. Aliquots of the brain homogenate (300 μL) were loaded into the donor side, and 500 μL of PBS into the receiver side of the dialyzer. Upon completion of dialysis that was performed at 37 °C for 5.5 h with shaking, aliquots (100 μL) of samples taken from both donor and receiver sides were added to an equal volume of PBS or corresponding blank matrix, respectively, followed by three times the volumes of acetonitrile containing internal standard. These samples were then vacuum filtered using a Strata Impact 96-well protein precipitation plate (Phenomenex). An aliquot of the filtrate was injected for LC-MS/MS analysis.

### Matrix-assisted laser desorption/ionization-time of flight (MALDI-TOF) mass spectrometry

Immunoprecipitation of carboxyl-terminally truncated Aβ peptides from 4 mL of H4 cell media was conducted using the anti-Aβ antibody 6E10 (epitope 3–8 of Aβ; Covance, Inc., Dedham, MA, USA) or 4G8 (epitope 18–22 of Aβ; Covance, Inc., Dedham, MA, USA) coupled to magnetic beads as described elsewhere [35]. After elution of the immunoprecipitated Aβ peptides, the detection was performed on an UltraFlextreme MALDI-TOF/TOF instrument (Bruker Daltonics, Bremen, Germany).

### Extraction of APP-derived tri-, tetra-, and pentapeptides from living cultured cells

The extractions were performed according to the methods described by Okochi et al. [36]. Briefly, human embryonic kidney (HEK) cells stably expressing APPsw and PS1 derivatives were cultured to confluence in 10-cm dishes and the media replaced on the day of the experiment. Cells were treated with FRM-36143 in the presence of proteasome inhibitors (1 mM lactacystin, 100 nM MG262, and 1 mM epoxomicin) for 1 h. Conditioned media were collected and cells were washed with ice-cold PBS and then immediately boiled for 2 min. A monitoring peptide (IVTL) and protease inhibitors were then added to the boiled samples, which were sonicated for 5 s three times and centrifuged at 100,000 × *g* for 1 h. The resultant supernatants were precipitated with TCA on ice for 15 min, centrifuged at 100,000 × *g* for 1 h, and filtered before being subjected to LC-MS/MS analysis to measure the tri-, tetra-, and pentapeptides as described elsewhere [37].

### Modeling and simulation

A population pharmacokinetic/pharmacodynamic (PK/PD) analysis was performed to obtain maximal efficacy (*E*_*max*_) and concentration leading to 50 % of *E*_*max*_ (EC_50_) using the Phoenix® NLME™ (NonLinear Mixed Effect) program (version 6.3, Certara USA, Inc., St. Louis, MO). The goodness of fit was assessed based on visual inspection of curve fitting, Akaike’s information criterion (AIC), and precision of parameter estimation.

## Results

### FRM-36143 modulates APP processing in vitro and does not affect Notch processing

**Fig. 1:**
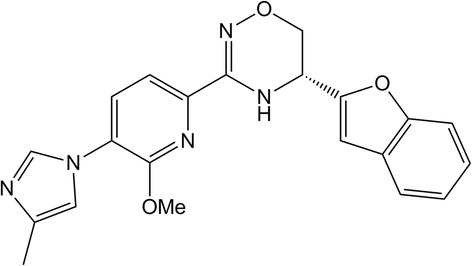
Structure of FRM-36143

**Fig. 2:**
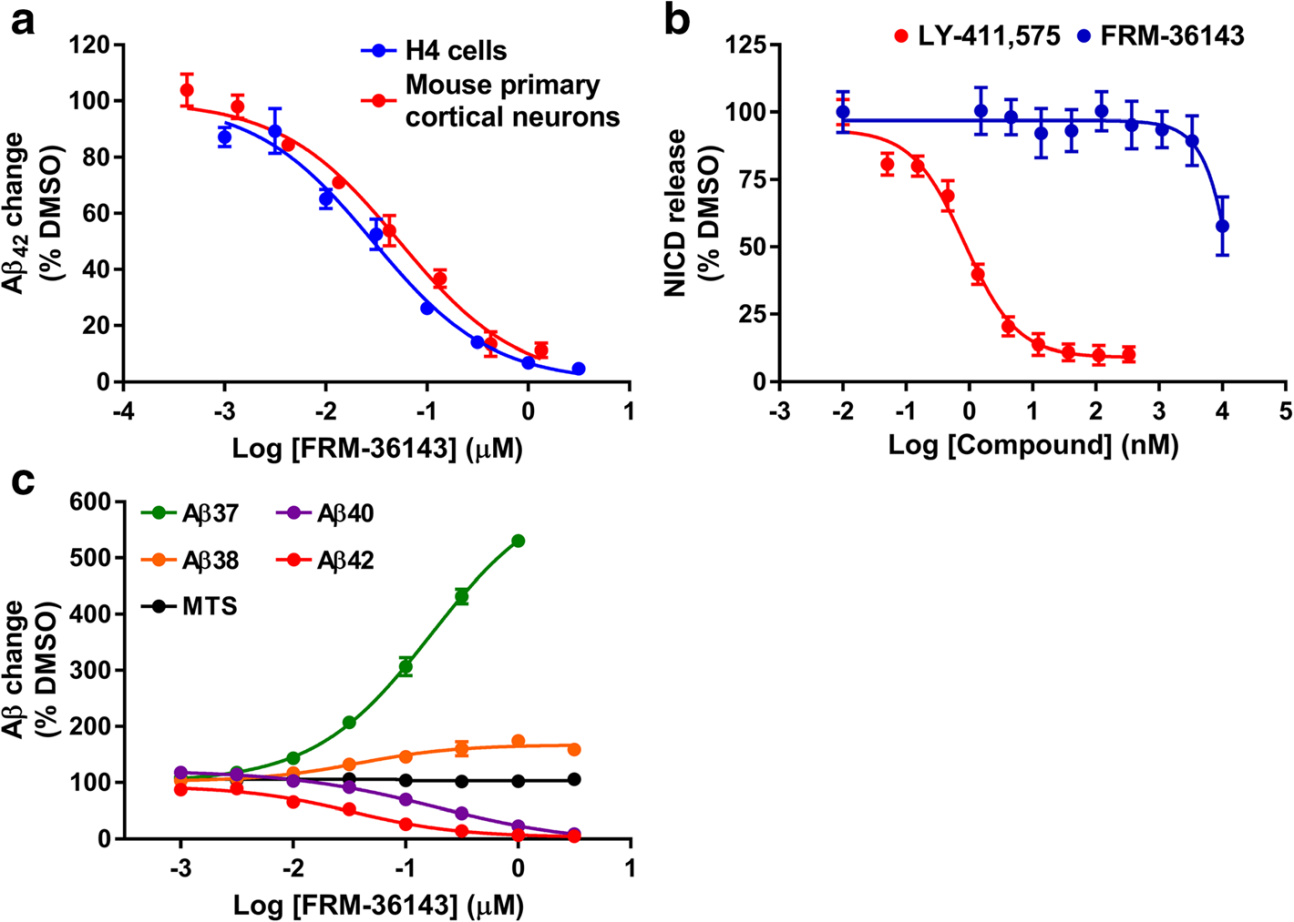
FRM-36143 is a potent GSM in vitro. **a** FRM-36143 potency on Aβ_42_ in H4 cells (35 nM) compared to mouse primary cortical neurons (53 nM). Data represent mean ± SEM of 2–5 experiments run in duplicate. **b** Potency of FRM-36143 (>10 μM) compared to the potent GSI, LY-411,575 (0.8 nM) on Notch processing. Data represent mean ± SEM of 2–5 experiments run in duplicate or triplicate. **c** Peptide profile from the media of H4 cells treated with FRM-36143 measured by ELISA. EC_50_ values: Aβ_37_ = 186 nM, Aβ_38_ = 38 nM, Aβ_40_ = 167 nM, Aβ_42_ = 35 nM

To first determine the potency of FRM-36143 (structure shown in Fig. 1), H4 cells stably expressing human wild-type (WT) APP751 were treated overnight with the compound, and Aβ_42_ levels were assessed from the conditioned media. Using this assay, it was determined that the EC_50_ of FRM-36143 for Aβ_42_ peptide modulation was 35 nM. The potency was then assessed in mouse primary cortical neuron cultures, where the EC_50_ for Aβ_42_ was determined to be 53 nM (Fig. 2a), which is within the assay variability and similar to the results from H4 cells.

To ascertain the GSM nature of FRM-36143, we verified that it did not prevent the processing of Notch. We compared its effect to that of the highly potent GSI, LY-411,575. As shown in Fig. 2b, FRM-36143 had no effect on Notch processing (IC_50_ > 10 μM), as observed by the lack of inhibition in the reporter assay at relevant concentrations. On the other hand, LY-411,575 was extremely potent at blocking Notch cleavage with a measured IC_50_ of 0.9 nM.

**Fig. 3:**
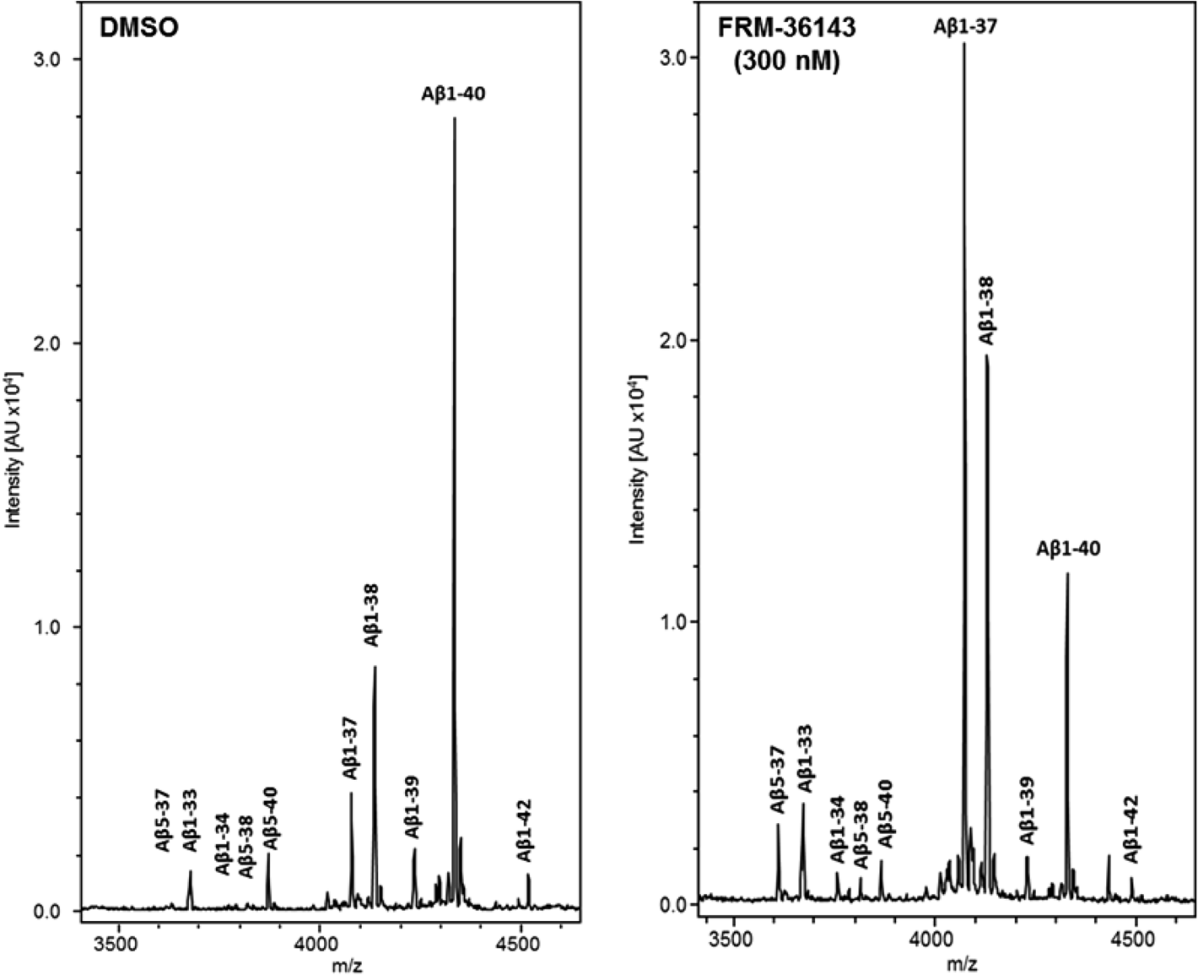
FRM-36143 affects multiple Aβ isoforms to different extents. Mass spectrometry traces showing the peptide profile in H4 cell culture media after overnight treatment with DMSO (*left*) or 300 nM FRM-36143 (*right*). Measurements are semi-quantitative. Note that peak height is relative for each Aβ isoform, and it should not be compared between peptides since the ionization efficiency and hydrophobicity might be different for each Aβ isoform

In order to assess the effect of FRM-36143 on other Aβ peptides, the APP751 H4 cells were again treated overnight with FRM-36143 and Aβ levels from the conditioned media assessed by ELISA. We determined the EC_50_ values to be 35 nM for Aβ_42_, 186 nM for Aβ_40_, 38 nM for Aβ_38_, and 167 nM for Aβ_37_ (Fig. 2c). From the same experiment, conditioned medium from H4 cells treated with 300 nM of the compound was analyzed by immunoprecipitation followed by mass spectroscopy as described elsewhere [35]. As depicted in Fig. 3, there was a large increase in the levels of Aβ_37_-derived peptides (Aβ_1–37_ and Aβ_5–37_) as well as a more modest increase in Aβ_1–33_, Aβ_1–34_, and Aβ_38_-derived peptides (Aβ_1–38_ and Aβ_5–38_). As expected, levels of Aβ_1–42_ were decreased by FRM-36143, and so were the levels of Aβ_1–39_ and Aβ_40_-derived peptides (Aβ_1–40_ and Aβ_5–40_). The extent of the change for each peptide is reported in Table 1.

**Table 1:** Normalized AUC and relative percentage change versus DMSO for each peptide measured by mass spectrometry

	Aβ_5–37_	Aβ_5–38_	Aβ_5–40_	Aβ_1–33_	Aβ_1–34_	Aβ_1–37_	Aβ_1–38_	Aβ_1–39_	Aβ_1–40_	Aβ_1–42_
DMSO	0.4	1	3	1.8	0.4	5.8	11.9	3.5	38.9	1.9
FRM-36143	1.9	1.5	1.2	2.9	0.5	23.6	15	1.6	10	0.4
Percentage change (vs. DMSO)	475	150	40	161	125	407	126	46	26	21

**Fig. 4:**
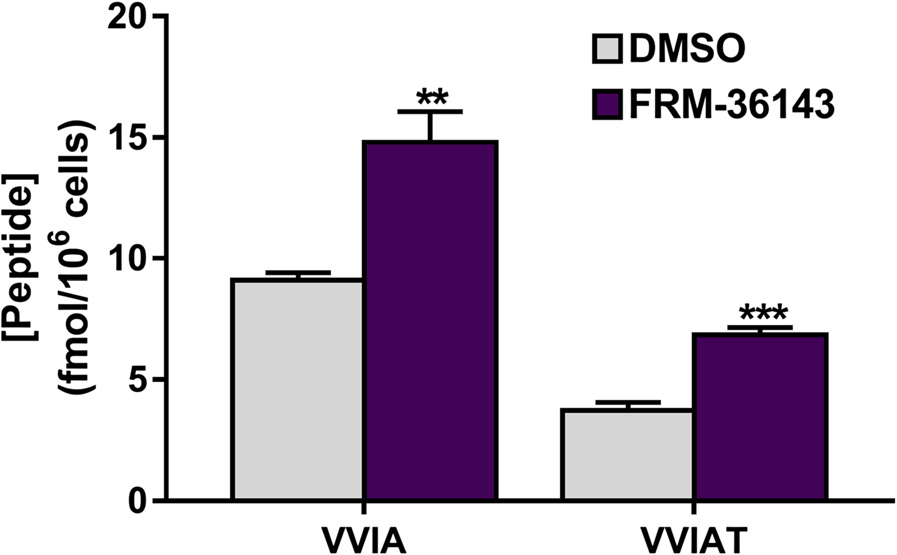
FRM-36143 increases the fourth cleavage step of gamma secretase. Using the step-wise cleavage model for Aβ cleavage, the generation of the tetrapeptide VVIA (Aβ_42_ → Aβ_38_) and of the pentapeptide VVIAT (Aβ_43_ → Aβ_38_) is increased by FRM-36143 in HEK cells expressing WT presenilin. Data represent mean ± SEM of *n* = 4. Unpaired *t* test: ** *p* < 0.01, *** *p* < 0.001

Finally, the levels of Aβ peptides in conditioned media and of intracellular small peptides produced from γ-secretase cleavage were measured from HEK cells expressing APPsw following 2 h of treatment with 1 μM of FRM-36143. As shown in Table 2, FRM-36143 significantly increases Aβ_38_ and decreases Aβ_40_ and Aβ_42_ while not changing Aβ_total_ (Aβ_38_ + Aβ_40_ + Aβ_42_). As for the production of small peptides, FRM-36143 increases the production of VVIA and VVIAT, which are the tetra- and pentapeptides derived from the fourth cleavage step by GS processing Aβ_42_ to Aβ_38_ (48→45→42→38) and Aβ_43_ to Aβ_38_ (49→46→43→38), respectively (Fig. 4).

**Table 2:** Aβ concentration (pM) in the media of HEK cells transfected with WT presenilin. Aβ_total_ is the result of the addition of Aβ_38_, Aβ_40_, and Aβ_42_

	DMSO	FRM-36143	*p* value
Aβ_total_	4925 ± 185	5130 ± 92	0.161
Aβ_38_	750 ± 62	1548 ± 39	4.8E-05
Aβ_40_	4021 ± 132	3477 ± 120	6.2E-03
Aβ_42_	154 ± 2	105 ± 2	6.5E-06

### Aβ-lowering effect of FRM-36143 in rodent brain and CSF

**Fig. 5:**
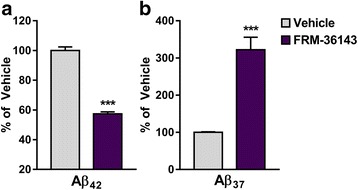
FRM-36143 is efficacious at modulating Aβ peptides in the mouse brain. Brain Aβ_42_ (**a**) and Aβ_37_ (**b**) are reported as percentage change from the vehicle-treated animals. Aβ_42_ = 43 % decrease, Aβ_37_ = 3.2-fold increase. Data represent mean ± SEM of 7–12 animals per group. Unpaired *t* test: *** *p* < 0.001

**Fig. 6:**
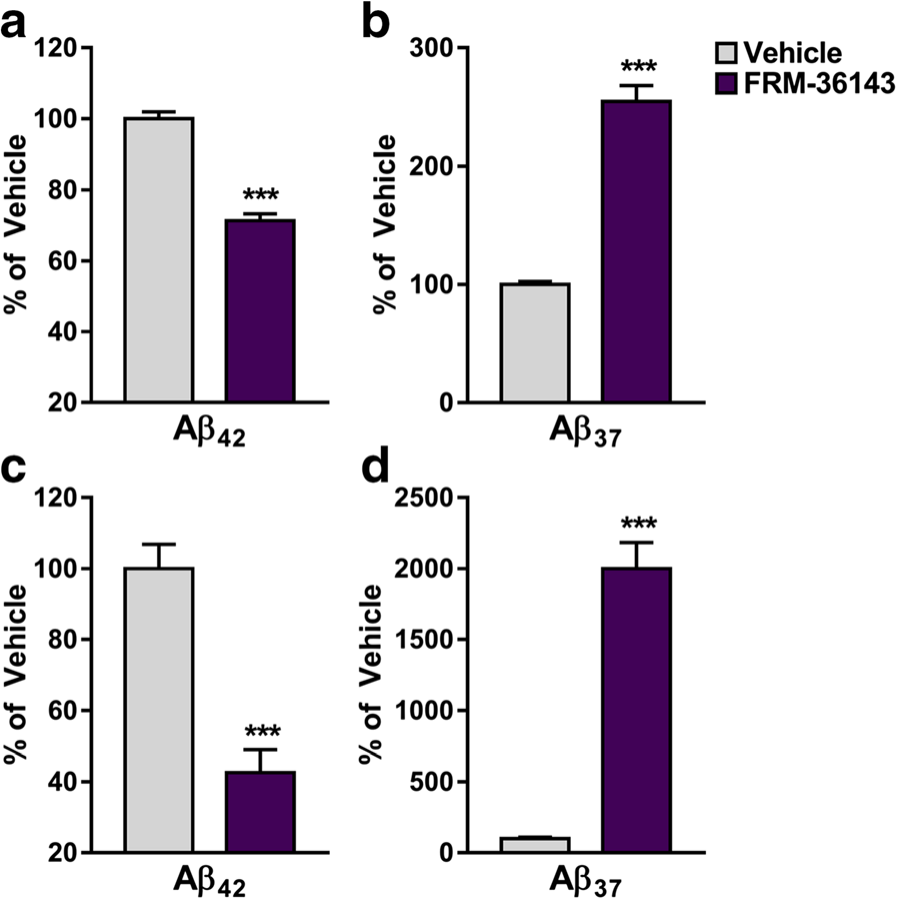
FRM-36143 is efficacious at modulating Aβ peptides in the rat brain and CSF. Brain (**a**, **b**) and CSF (**c**, **d**) Aβ peptide changes are reported as percentage change from the vehicle-treated animals. FRM-36143 led to a reduction of 30 % and 58 % reduction of Aβ_42_ in brain (**a**) and CSF (**c**), respectively. This was accompanied by increases of 2.5-fold and 20-fold of Aβ_37_ in the brain (**b**) and CSF (**d**). Data represent mean ± SEM of 7 animals per group. Unpaired *t* test: *** *p* < 0.001

**Fig. 7:**
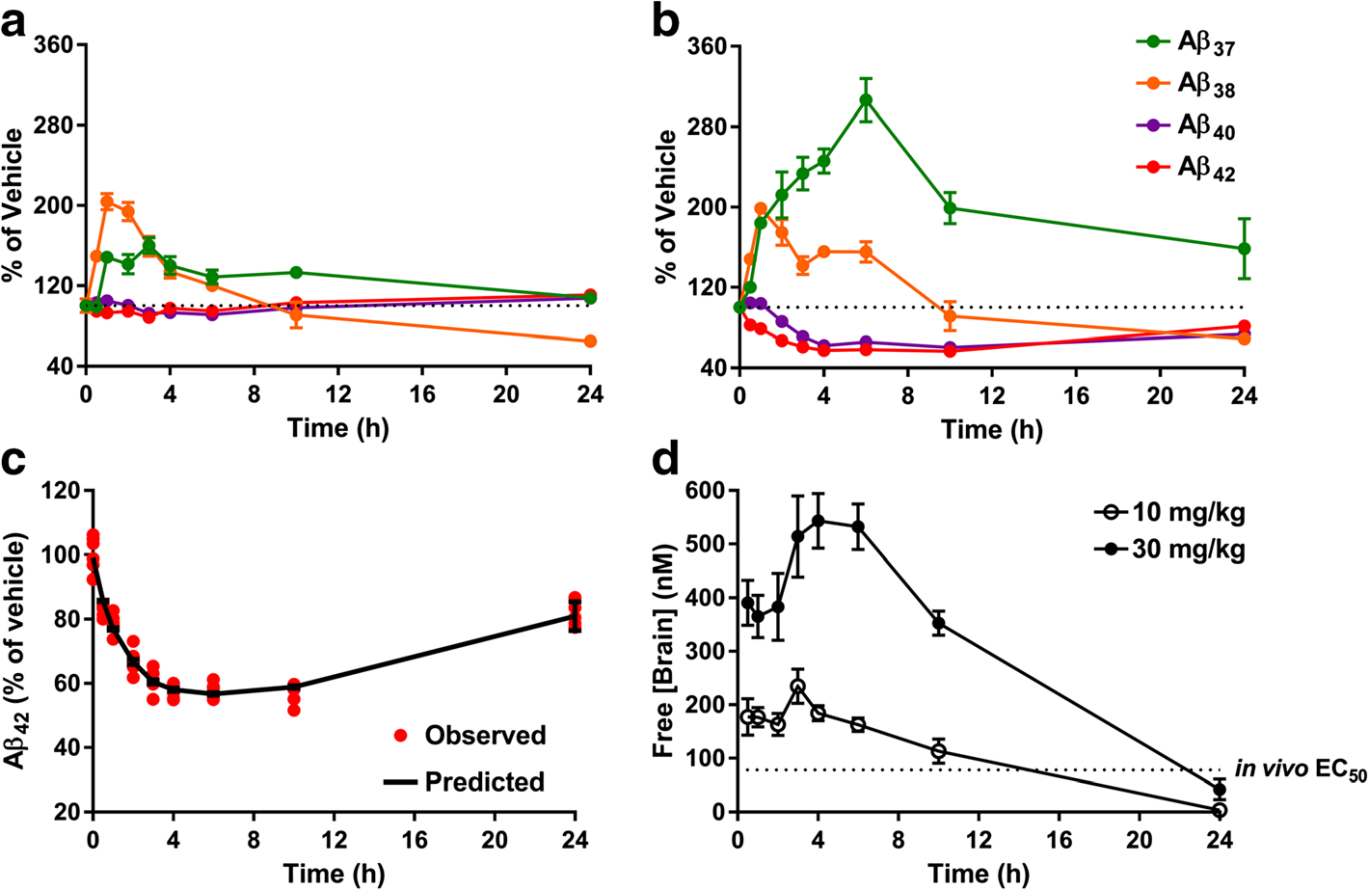
FRM-36143 induces a sustained change in mouse brain Aβ peptides. Time course of Aβ peptide profile changes in the brain at (**a**) 10 mg/kg and (**b**) 30 mg/kg FRM-36143. **c** pharmacokinetic/pharmacodynamic (*PK/PD*) modeling of the 30 mg/kg dose. **d** Unbound brain exposure of FRM-36143 at 10 and 30 mg/kg. Data represent mean ± SEM of 5–8 animals per group

In order to assess the in vivo efficacy of FRM-36143, we opted to use WT animals to avoid any potential confounding factor associated with overexpression of APP. We first treated mice with a single oral dose of 30 mg/kg and measured the pharmacodynamic markers Aβ_37_ and Aβ_42_ 6 h post-treatment. This time point was chosen to eliminate compounds with a half-life too short to induce a pharmacodynamic change in the brain. As shown in Fig. 5, FRM-36143 reduced Aβ_42_ by 43 % (Fig. 5a) and increased Aβ_37_ 3.2-fold (Fig. 5b) in mouse brain. We then tested the compound in rats to compare its effect in brain and CSF. We observed a 30 % Aβ_42_ reduction in the brain (Fig. 6a) accompanied with a 2.5-fold increase in Aβ_37_ (Fig. 6b). In CSF, the effect was greater, with a 58 % reduction in Aβ_42_ (Fig. 6c) and a 20-fold increase in Aβ_37_ (Fig. 6d). Finally, in order to determine the in vivo Aβ_42_ EC_50_ of FRM-36143, we performed a time course study in mice. We treated the animals with oral doses of 10 and 30 mg/kg and measured brain levels of Aβ_37_, Aβ_38_, Aβ_40_, and Aβ_42_ at multiple time points (0.5, 1, 2, 3, 4, 6, 10, 24 h). While 10 mg/kg induced an increase in Aβ_37_ and Aβ_38_, it did not reduce Aβ_40_ and Aβ_42_ to a great extent (Fig. 7a). On the other hand, the 30 mg/kg dose led to 40 % and 45 % peak reductions of Aβ_40_ and Aβ_42_, respectively, with a calculated AUC_0→24h_ of 31 % for Aβ_40_ and 34 % for Aβ_42_ (Fig. 7b). Peak increases of 3.1-fold and 2-fold were observed for Aβ_37_ and Aβ_38_, respectively. Using the exposure shown in Fig. 7d, we performed population PK/PD model fitting for the effect on Aβ_42_ and calculated the *E*_*max*_ to be 0.51 (CV: 15 %) and the in vivo EC_50_ to be 78 nM (2.5–97.5 % CI; 4.6–151 nM; unbound plasma concentration). Given the very small effect of the GSM at 10 mg/kg, the model did not capture the data well enough to be included in the parameters estimation; hence, only the 30 mg/kg data were used (Fig. 7c).

**Fig. 8:**
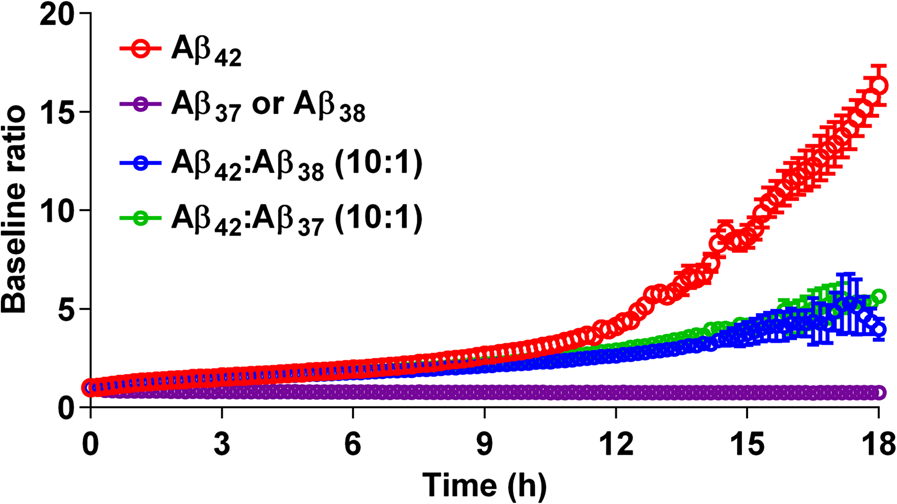
Short Aβ peptides slow down aggregation of Aβ_42_ in vitro. Co-incubation of the shorter peptides Aβ_37_ or Aβ_38_ with Aβ_42_ in a ratio of 1:10 reduces aggregation of Aβ_42_ by more than threefold after 18 h under the assay conditions. Aβ_37_ and Aβ_38_ do not aggregate on their own. Data represent mean ± SEM of two experiments run in triplicate

The increases in shorter peptides Aβ_37_ and Aβ_38_ led us to investigate how they affected the aggregation potential of Aβ_42_. As shown in Fig. 8, the short Aβ peptides did not aggregate under our assay conditions, whereas Aβ_42_ showed a >15-fold increase in aggregates over time measured as thioflavin T fluorescence. Mixing Aβ_37_ or Aβ_38_ with Aβ_42_ at a ratio of 1:10 (short to long peptide) reduced Aβ_42_ aggregation by more than threefold, and larger ratios of short to long Aβ peptides completely prevented aggregation (data not shown).

### FRM-36143 does not affect cholesterol metabolism

**Fig. 9:**
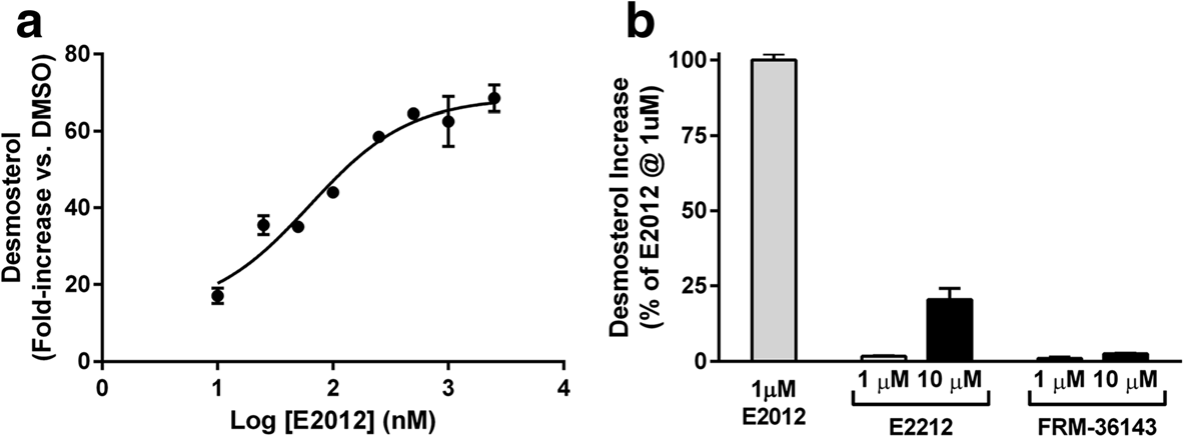
FRM-36143 does not inhibit DHCR24. Desmosterol change from DMSO was measured in presence of the positive control E2012, the negative control E2212, and FRM-36143. **a** Dose–response inhibition of DHCR24 by E2012 (IC_50_ = 63 nM; desmosterol induction at 1 μM ~ 60-fold). **b** Desmosterol fold increase for the negative control E2212 and FRM-36143 as compared to the response of E2012 at 1 μM. Data represent mean ± SEM of one or two experiments run in duplicate

The GSM E2012 was reported to inhibit 24-dehydrocholesterol reductase (DHCR24), the enzyme responsible for the last step in cholesterol biosynthesis, catalyzing the reduction of desmosterol to cholesterol [33]. In order to test if FRM-36143 had the same off-target activity, we developed an in vitro assay to monitor the accumulation of desmosterol in cells. Using E2012 as the positive control, we determined that its IC_50_ for DHCR24 was 63 nM and that it maximally inhibited DHCR24 at 1 μM (Fig. 9a). We then compared the effect of FRM-36143 relative to 1 μM E2012 and found that it only led to a desmosterol accumulation of 1.1 % at 1 μM and 2.6 % at 10 μM (Fig. 9b). This result suggests that FRM-36143 does not have off-target activity at DHCR24.

### FRM-36143 reverses the effect of FAD PS1 mutations

**Fig. 10:**
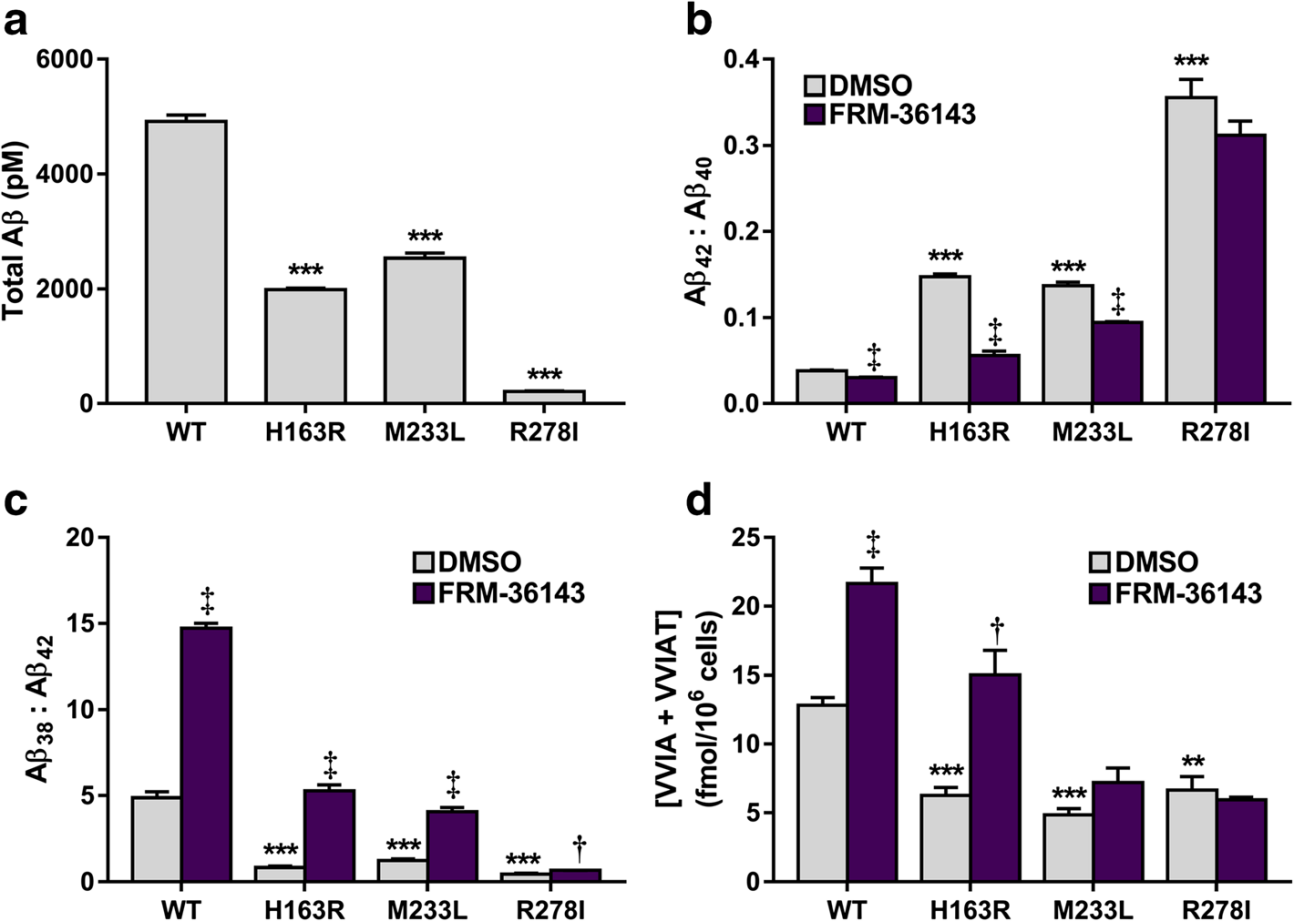
FRM-36143 reverses the effects of FAD presenilin mutants. **a** Total Aβ secreted by HEK cells expressing WT or mutant PS. Presenilin mutants cause a partial loss of function of γ-secretase leading to a decreased secretion of total Aβ peptides. **b** Presenilin mutations increase the Aβ_42_:Aβ_40_ ratio, which is reversed by FRM-36143. Presenilin mutations decrease the fourth cleavage cycle of γ-secretase as measured by the Aβ_38_:Aβ_42_ ratio (**c**) or the intracellular production of the peptides VVIA and VVIAT (**d**), both of which are reversed by FRM-36143. Data represent mean ± SEM of *n* = 4. Two-tailed unpaired *t* test: ** *p* < 0.001 and *** *p* < 0.0001: mutants vs. WT, ‡ *p* < 0.0001: FRM-36143 vs. DMSO, † *p* < 0.01: FRM-36143 vs. DMSO

We wanted to verify the potential of FRM-36143 to reverse the effect of FAD PS1 mutations towards APP processing. We tested the effect of the compound in cell lines overexpressing APPsw together with different forms of FAD PS1 mutations (H163R, M233L, or R278I). As shown in Fig. 10a, the three PS1 mutants produced less Aβ overall compared to the WT. PS1 mutations are associated with increased Aβ_42_:Aβ_40_ ratios, which are also observed here (Fig. 10b), as well as a decrease in the fourth cleavage step of GS as measured by the ratio of Aβ_38_:Aβ_42_ (Fig. 10c) or the production of the small peptides VVIA (Aβ_42_→Aβ_38_) and VVIAT (Aβ_43_→Aβ_38_) (Fig. 10d). In almost all cases, FRM-36143 is able to reverse the effect of the mutations studied. The R278I mutant seems to be more resistant to the effect of the compound. Its Aβ_42_:Aβ_40_ ratio remains unchanged by the treatment and, while FRM-36143 produced a significant increase on the fourth cleavage step as measured by the Aβ_38_:Aβ_42_ ratio, it remains much more subtle than for the two other mutants.

## Discussion

The genetic evidence linking APP and PS mutations to AD strongly supports the idea that APP processing and the resulting Aβ peptides are directly linked to the neuropathological changes observed in AD. It has been suggested that Aβ could mediate toxicity either by driving the disease pathophysiology or by triggering downstream events [38]. A protective mutation in APP has also been reported to delay disease onset and has been linked to decreased production and aggregation of Aβ [39, 40]. Targeting the production and/or degradation of Aβ are thus some of the best rationales to delay the onset of symptoms. γ-Secretase, with its core catalytic component presenilin, has been a prime target to address the amyloid pathology. Unfortunately, early efforts at developing GSIs were met with clinical failure. These compounds showed no substrate selectivity, which led to undesirable side effects such as skin cancers, weight loss, infections, and even exacerbated memory decline [25, 27, 28]. After the discovery by Weggen et al. [31] that a subset of NSAIDs could selectively lower Aβ_42_ without affecting the initial cleavage step by GS (ε cleavage), a lot of research was dedicated to finding new generations of compounds that could mediate similar effects [20, 32]. In our quest to discover GSMs that satisfy all known empirical rules of good central nervous system (CNS) drug-like properties [41–43], we synthesized FRM-36143 (Bursavich MG, Harrison BA, Costa DE, Hodgdon HE, Freeman EA, Hrdlicka LA, Kapadnis S, Moffit J, Murphy DA, Patzke H, Tang C, Wen M, Burnett DA, Koenig G, Blain JF. Design, synthesis and evaluation of a novel series of oxadiazine Gamma Secretase Modulators for familial Alzheimer’s disease, Submitted). Characterization of this compound proved it to be an excellent modulator of γ-secretase with potential for treating FAD.

We evaluated the potential of FRM-36143 to reduce Aβ_42_ levels in cells and found that it did with an EC_50_ of 35–53 nM. When looking at other peptides, we found the EC_50_ for Aβ_38_ (38 nM) to be similar to that of Aβ_42_, whereas the EC_50_ values for Aβ_37_ and Aβ_40_ were similar to each other (167 and 186 nM, respectively) but fivefold less potent when compared to Aβ_42_. This observation fits well the basic tri/tetra-peptide model put forward by Ihara and colleagues in which Aβ_38_ is mainly derived from Aβ_42_ and Aβ_37_ from Aβ_40_ [37, 44, 45]. This model has since been refined to include multiple routes by which the different Aβ isoforms could be produced [46]. It has been suggested that GSMs help promote the fourth cleavage cycle of GS as exemplified by the increased ratio of Aβ_38_:Aβ_42_ [17]. Here we show that FRM-36143 increases that fourth cycle as measured by the intracellular accumulation of the cleavage products VVIA (Aβ_42_→Aβ_38_) and VVIAT (Aβ_43_→Aβ_38_) as well as an increase in the Aβ_38_:Aβ_42_ ratio.

Monitoring of Aβ peptides by mass spectrometry shows that Aβ_39_ and longer peptides were reduced by FRM-36143, whereas Aβ_38_ and shorter peptides were increased, similarly to what was measurable by ELISA. These changes also hold true for the N-terminally truncated forms Aβ_5-x_. These findings are important, as it is well accepted that Aβ_42_ is the most aggregation-prone peptide, forming the core of amyloid plaques [10] and, as we show in this study, the shorter forms like Aβ_37_ and Aβ_38_ can prevent its aggregation. Moreover, the reduction in Aβ_5–40/42_ is significant, since N-terminally truncated Aβ_40/42_ were shown to be even more aggregation-prone than Aβ_42_ [47]. Interestingly, it was reported that BACE inhibitors increase the levels of Aβ_5–40_ and Aβ_5–42_ in multiple models [48] as well as in patient CSF [49], suggesting that GSMs might be a preferred treatment strategy if there is concern about N-terminally truncated peptides.

We show that FRM-36143 induced increases of Aβ_37_ with the largest change from baseline of any Aβ peptide (fourfold to fivefold). This result is likely due to the fact that Aβ_37_ is mainly derived from Aβ_40_, which is the most prevalent peptide (~10-fold higher than Aβ_37_ and Aβ_42_). Because of this, we believe Aβ_37_ would make an excellent biomarker for the clinic, as its levels are not changed in AD patient CSF [50], and it has the largest dynamic range of all changes observed. On the other hand, Aβ_42_ levels are already reduced in AD CSF, an observation that could confound the effect of the GSM.

The first cleavage step by GS (ε cleavage) is crucial for processing both Notch [51] and APP [9]. Inhibition of Notch processing was one of the main problems reported from GSI clinical trials, where side effects are thought to have resulted in part from Notch-related toxicity [25, 27, 28]. Moreover, GS needs to perform a similar ε cleavage; otherwise, its substrate C99 accumulates in the membrane and has been reported to be toxic [52, 53]. The accumulation of C99 has even been suggested as an explanation for the memory decline observed in the semagacestat trial [30]. We thus verified that FRM-36143 did not have the potential to impede the release of the Notch intracellular domain (NICD). We compared its activity to the GSI LY-411,575 in a Notch reporter assay and, as expected, Notch processing was not affected by FRM-36143 (IC_50_ > 10 μM), while the GSI was extremely potent at blocking the release of NICD (IC_50_ = 0.9 nM).

Compounds with the desired effect on Aβ_42_ in vitro were tested in animals to assess their pharmacokinetic (PK) and pharmacodynamic (PD) properties. We first assessed the effect of a 30 mg/kg oral dose of FRM-36143 after 6 h in mouse brain and found that it produced a 43 % reduction in Aβ_42_ accompanied by a 3.2-fold increase in Aβ_37_ at an unbound brain concentration of 427 nM. Interestingly, when we dosed at 90 mg/kg we did not achieve significantly higher exposure or efficacy, suggesting that compound absorption was rate limiting (data not shown). We then wanted to compare the extent of efficacy between brain and CSF, as the latter compartment would be used in the clinic to measure compound efficacy. Dosing rats with 30 mg/kg FRM-36143 led to 30 % and 58 % Aβ_42_ reductions in the brain and CSF, respectively, 6 h post-dose. It also increased Aβ_37_ by 2.5- and 20-fold in those compartments. Using brain tissue binding, we calculated the unbound brain exposure to be 156 nM, whereas it was measured at 122 nM in the CSF, suggesting a good concordance between the two measures. We found Aβ levels to be low in rats, which put most of the Aβ_37_ values in the vehicle group close to the detection limit of the assay. Because of this limitation, it is possible that increases were overestimated. Despite this issue, it is clear that changes are much larger in CSF compared to brain for both peptides, which is in line with a faster clearance rate of Aβ peptides from the CSF [54].

In order to determine the in vivo EC_50_ of FRM-36143 and verify the in vitro–in vivo correlation, we performed a time course in mice at two different doses. Modeling brain Aβ_42_ reduction produced an EC_50_ of 78 nM, which correlates well with the in vitro EC_50_ of 35 to 53 nM measured in H4 cells and primary neurons. The model also allowed for an estimation of the maximal brain Aβ_42_ reduction (*E*_*max*_) achievable with FRM-36143, which was calculated to be 51 %. Surprisingly, we did not observe any efficacy at the 10 mg/kg dose on Aβ_42_ reduction, even though we were reaching exposure levels (unbound brain Cmax = 235 nM) well above the EC_50_ for more than 6 h. After testing multiple compounds in this class, we empirically observed that in order to achieve significant in vivo efficacy we had to reach unbound concentrations in the brain that approached the EC_90_ for these compounds. FRM-36143 has an EC_90_ of 383 nM, which is higher than the Cmax achieved at 10 mg/kg.

The potent GSM E2012 was reported to cause lenticular opacity in animals [33]. Because this off-target toxicity takes 12–14 weeks to appear in animals, we developed an assay that allowed us to screen against it in vitro. Eisai identified DHCR24 as being involved in the development of cataracts in animals. They showed that E2012 inhibited the last step in the cholesterol biosynthetic pathway leading to the accumulation of the precursor, desmosterol, in the lens and in plasma as soon as 24 h after treatment. They had also identified the compound E2212 that did not have this off-target activity [55]. Using this information, we screened for the accumulation of desmosterol in HepG2 cells and found that FRM-36143 was even less potent than the negative control E2212. This result clearly showed that this off-target activity is avoided by FRM-36143.

Finally, PS mutations have been reported to decrease the efficiency of the fourth cleavage step of APP, leading to a decreased Aβ_38_:Aβ_42_ ratio [17] as well as an increased Aβ_42_:Aβ_40_ ratio [56]. GSMs were shown to increase the processivity of the enzyme complex [36], and we confirmed this using FRM-36143. Of the three PS1 mutants we analyzed (H163R, M233L, R278I), all showed a reduction in total Aβ production compared to WT PS1, highlighting an overall GS partial loss of function [57]. This loss of function was also accompanied by an increased Aβ_42_:Aβ_40_ ratio and a decreased Aβ_38_:Aβ_42_ ratio compared to WT PS1. FRM-36143 was able to reverse the effect of the mutations as measured by the ratios as well as increase the production of the fourth cleavage cycle products VVIA (Aβ_42_→Aβ_38_) and VVIAT (Aβ_43_→Aβ_38_). The R278I mutant seemed to be resistant to the GSM and, interestingly, it was reported that this mutation impaired endoproteolysis of PS1, which causes a selective increase in Aβ_43_ [58, 59]. Moreover, patients carrying this mutation presented with language impairments and did not meet the AD clinical criteria [60].

## Conclusions

In conclusion, we described a novel molecule, FRM-36143, which possesses all the characteristics of a GSM in terms of Aβ modulation, does not inhibit Notch processing, and is devoid of activity against DHCR24. Moreover, the design of FRM-36143 limits its lipophilic nature, enhancing its very good CNS drug-like properties (Bursavich MG, Harrison BA, Costa DE, Hodgdon HE, Freeman EA, Hrdlicka LA, Kapadnis S, Moffit J, Murphy DA, Patzke H, Tang C, Wen M, Burnett DA, Koenig G, Blain JF. Design, synthesis and evaluation of a novel series of oxadiazine Gamma Secretase Modulators for familial Alzheimer’s disease, Submitted) and makes it an excellent candidate for further characterization. Because FAD mutations in PS have been shown to cause a partial loss of function of γ-secretase [17, 56, 57], it was rewarding to see that FRM-36143 was able to reverse the effect of most PS mutations tested. Given this result, we suggest that targeting a patient population with this genetic defect would be the most straightforward approach to testing the efficacy of a GSM in the clinic.

In sporadic AD, the amyloid hypothesis is not linked to any specific genetic defect; thus, treatment with BACE inhibitors or monoclonal antibodies relies on an ascribed role for Aβ in the disease progression. In contrast, PS mutations underlie the genetic defect in FAD, causing the earlier disease onset. Our data, together with that of others [61], suggest that GSMs can correct the partial loss of function of γ-secretase caused by many PS mutants. We thus believe that GSMs, like FRM-36143, have the potential to prevent the disease in patients with FAD when treatment starts early in the course of its development.

## Abbreviations

AD, Alzheimer’s disease; Aph-1, anterior pharynx-defective 1; APP, amyloid precursor protein; APPsw, amyloid precursor protein with a Swedish mutation; AUC, area under the curve; BACE, β-site APP-cleaving enzyme; Aβ, beta amyloid; CNS, central nervous system; CSF, cerebrospinal fluid; DHCR24, 24-dihydrocholesterol reductase; EC_50_, efficacious concentration producing a 50 % effect; *E*_*max*_, maximal effect; FAD, familial Alzheimer’s disease; f_u,b_, brain unbound fraction; GS, γ-secretase; GSI, γ-secretase inhibitor; GSM, γ-secretase modulator; Nct, nicastrin; NICD, Notch intracellular domain; NSAID, nonsteroidal anti-inflammatory drug; PD, pharmacodynamic; Pen-2, presenilin enhancer-2; PK, pharmacokinetic; PS, presenilin; WT, wild type
